# Neural Correlates of Direct Access Trading in a Real Stock Market: An fMRI Investigation

**DOI:** 10.3389/fnins.2017.00536

**Published:** 2017-09-29

**Authors:** GianMario Raggetti, Maria G. Ceravolo, Lucrezia Fattobene, Cinzia Di Dio

**Affiliations:** ^1^Centre for Health Care Management, School of Medicine, Università Politecnica delle Marche, Ancona, Italy; ^2^Department of Management, School of Economics, Università Politecnica delle Marche, Ancona, Italy; ^3^Department of Experimental and Clinical Medicine, School of Medicine, Università Politecnica delle Marche, Ancona, Italy; ^4^Department of Psychology, Università Cattolica del Sacro Cuore, Milan, Italy

**Keywords:** neuroeconomics, cognitive control, fMRI, financial risk taking, financial market, dorsolateral prefrontal cortex

## Abstract

**Background:** While financial decision making has been barely explored, no study has previously investigated the neural correlates of individual decisions made by professional traders involved in real stock market negotiations, using their own financial resources.

**Aim:** We sought to detect how different brain areas are modulated by factors like age, expertise, psychological profile (speculative risk seeking or aversion) and, eventually, size and type (Buy/Sell) of stock negotiations, made through Direct Access Trading (DAT) platforms.

**Subjects and methods:** Twenty male traders underwent fMRI while negotiating in the Italian stock market using their own preferred trading platform.

**Results:** At least 20 decision events were collected during each fMRI session. Risk averse traders performed a lower number of financial transactions with respect to risk seekers, with a lower average economic value, but with a higher rate of filled proposals. Activations were observed in cortical and subcortical areas traditionally involved in decision processes, including the ventrolateral and dorsolateral prefrontal cortex (vlPFC, dlPFC), the posterior parietal cortex (PPC), the nucleus accumbens (NAcc), and dorsal striatum. Regression analysis indicated an important role of age in modulating activation of left NAcc, while traders' expertise was negatively related to activation of vlPFC. High value transactions were associated with a stronger activation of the right PPC when subjects' buy rather than sell. The success of the trading activity, based on a large number of filled transactions, was related with higher activation of vlPFC and dlPFC. Independent of chronological and professional age, traders differed in their attitude to DAT, with distinct brain activity profiles being detectable during fMRI sessions. Those subjects who described themselves as very self-confident, showed a lower or absent activation of both the caudate nucleus and the dlPFC, while more reflexive traders showed greater activation of areas involved in strategic decision making.

**Discussion:** The neural correlates in DAT are similar to those observed in other decision making contexts. Trading is handled as a well-learned automatic behavior by expert traders; for those who mostly rely on heuristics, cognitive effort decreases, and transaction speed increases, but decision efficiency lowers following a poor involvement of the dlPFC.

## Introduction

According to the Efficient Market Theory (EMT), stock market prices should reflect the influence of all available information at a given time. The first of the three basic hypotheses of this theory assumes investors are rational when they use information; the second assumes that they maximize expected utility and the third that if some investors behave irrationally, equilibrium prices deviates, but only in the short-term, because of the offset effects due to the random actions of other irrational traders or to the trades of rational “arbitrageurs.” However, systematic violations from rational behavior and continuous stock market crashes reveal the poor level of investor's rationality questioning the validity of traditional assumptions. In recent decades several behavioral studies have shown the role played by insights, heuristics, impulses, and emotions in financial decision making, focusing, in particular, on the influence of agent's mood in stock market prices (Saunders, 1993; Hirshleifer and Shumway, 2003), of individual biases, such as myopic loss aversion (Benartzi and Thaler, 1995; Gneezy and Potters, 1997), disposition effect (Odean, 1998), and overconfidence in both trading activity (Barber and Odean, 2000; Gervais and Odean, 2001; Grinblatt and Keloharju, 2009), and managerial choices (Heaton, 2002; Malmendier and Tate, 2005), and so on. Recent studies have demonstrated the neural correlates of financial decision making. Researchers are shedding light on the neural mechanism related to risk, uncertainty and ambiguity (for a review see: d'Acremont and Bossaerts, 2008; Mohr et al., 2010; Burke and Tobler, 2011; Bach and Dolan, 2012). to the personal representation of expected reward (for a review see: Clithero et al., 2008; Schultz, 2008), to market bubbles (De Martino et al., 2013), to the nature of trader intuition (Bruguier et al., 2010), to lending decisions (Genevsky and Knutson, 2015), or to the origin of the disposition effect (Frydman et al., 2014).

Until now, few studies have been focused on the neural aspects of traders' behavior in financial decision making. These studies recorded both brain activity and behavioral data using different methods but most of them, and surely, those conducted with fMRI, referred to stock markets simulated in laboratory. The analyses were made using virtual money and often involved participants with a much lower level of expertise than actual practitioners. Arguably, the results obtained are different from those that could be collected during decisions assumed in a real scenario, when people use their own (real) money (Smith and Walker, 1993; Camerer and Hogarth, 1999; Hertwig and Ortmann, 2001; Harrison and Rutström, 2008; Hensher, 2010; Vlaev, 2012). Lo and Repin (2002) recorded physiological data to link emotions and decision making in traders. They found that deviations of price, spread, and return, and trend reversal of price and spread, elicited skin conductance responses and abrupted variations in body temperature, while the volatility in share prices was related with blood volume pulses; they collected a higher number of physiological responses among less experienced traders. Coates and Herbert (2008) analyzed saliva's sample of 17 traders for 8 days to explore the link between testosterone and cortisol levels with financial returns and financial uncertainty, respectively. Their findings revealed the ability of morning testosterone to predict traders' profitability while the increase in cortisol was associated to market volatility and trading results variability. Coates et al. (2009) analyzed the second-to-fourth digit length ratio of male traders observing that it predicts both long-term profitability and years of permanence in the business. Kuhnen and Knutson (2011) investigated the influence of affective states on risk preferences and trading decisions, revealing that negative stimuli, either endogenous or exogenous, promote risk averse decisions while positive ones induce people to risk seeking behavior. Frydman et al. (2014) recorded students' brain activity, through fMRI, during negotiations in a virtual stock market, confirming the realization utility hypothesis. Lima Filho et al. (2015), using EEG, highlighted the involvement of cortical areas and their interconnections during traders' decision-making processes: they revealed that purchase and sale orders trigger different neuronal circuits. Huber et al. (2015) explored the neural correlates of informational cascade in a hypothetical decision scenario that included the presence of two fictitious traders. Results revealed that people tended to overrate private information as compared with social information and that when the former is conflicting with the latter, activations of the brain regions related to risk and uncertainty are detected.

In this study, we aimed at adding value in the literature by overcoming the limitation of artificial contexts and we sought to investigate the neural correlates of financial decision making considering a sample of professional financial traders who operated in the real Stock Market Exchange, using their own financial resources, while lying in a fMRI scanner. We were especially interested in unveiling the relationship between the activation of brain areas in the time periods preceding any decision, and the multiple demographical and contextual factors, supposed to influence decision making; moreover, we wanted to study such relationship in a scenario as much real as possible, to enhance motivation and arousal.

## Methods

### Participants and design

Subjects were healthy, right-handed males that operated as professional traders. In particular, participants were *intra-day* traders—who open and close a position in the same trading day—and *scalpers*—who hold positions for very short time intervals and aim at making profit from bid-ask spreads. Participants were recruited through an advertisement circulated by IW Bank in Milan. The original sample was composed of 22 subjects; two of them were not considered in the analysis because of their poor performance during the trading sessions. Therefore, all data refer to a final sample of 20 subjects. They were aged 27–51 years (mean: 40.3 ± 7.8) and declared 5–15 years trading experience (mean: 9.9 ± 2.3). They agreed to participate in the study and use their own personal bank-account at IW-Bank agency in Milan. Subjects suffering from claustrophobia or any other contraindication to be exposed to magnetic fields were excluded. A behavioral questionnaire was distributed to traders 1 month after the fMRI session, in order to look for the following personal and professional aspects:

- Relevance of trading activity on the annual overall personal income;
- Preferred financial markets and products;
- Type, volume and frequency of transactions executed in a usual trading session;
- Surplus, deficit, or break-even, reached in the last year of trading activity.
- Behavioral approach to *trading (whether* methodical and respectful of traditional operative rules, OR creative and influenced by mood)
- Perceived emotional involvement during the three following phases: (1) data and information processing, (2) operative decision, (3) negotiation conclusion
- Personal vision of the financial market, as a “network of professional competitors to beat” OR as a “source of information and data to process for profit.”

We avoided administering the questionnaire in close temporal contiguity with the fMRI session, as we believed that this would have influenced either the trading behavior, in case of questionnaire filled before the experiment, or the answers to the interview, in the opposite case. The questionnaire is available in its Italian version upon request to the authors.

All the answers collected offered a psychological description of the traders' professional behavior, which was then compared to the neural activation associated with decision making processes.

All traders except one claimed to have had a profitable trading activity in the previous year. Twelve subjects declared that trading was their only job and income source (they normally negotiate in a continuous and exclusive way), while the others considered it as a source of income integration.

Each trader selected *only one security* (they selected STM—STM Microelectronics, CPR-Campari, 6EZO, ESZO, BP-Banco Popolare, Minifib, Unicredit, SOPAF, TIS-Tiscali, and E7ZO). The *platforms* were *QuickTrade* (used by 9 traders), *EasyTrade* (6 cases), *Sphera* (2 cases), and *RealTick* (3 cases).

All subjects gave written informed consent in accordance with the Declaration of Helsinki. The protocol was approved by the local Ethics committee of the University of Parma.

### Experimental protocol

In order to implement an ecological reliable investigation protocol, allowing traders to negotiate in the real market staying in a fMRI scanner, several technical obstacles needed to be overcome.

**Figure 1:**
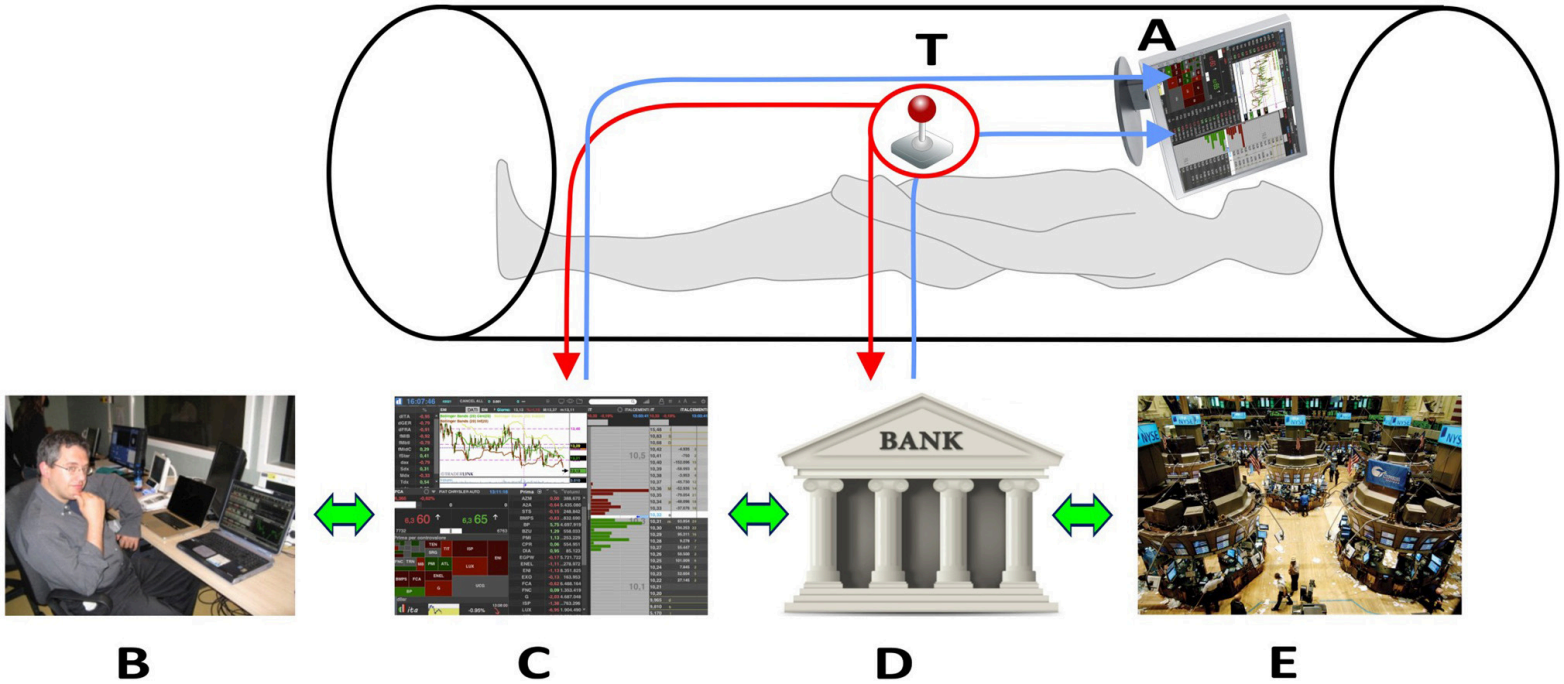
Technical solutions to collect neural signals in fMRI scanner. The trader in the fMRI scan look at the trading book (C) and market general trends (E) through the goggles (A). The trackball (T) allows the trader to operate in the scanner and insert his financial decisions, which directly affect his bank account (D). All the decisions are supervised by an expert (B) located outside the fMRI room. Written consent for the publication of this image was obtained from the subject represented in (B).

Figure 1 shows the innovative technical solutions adopted.

**Figure 2:**
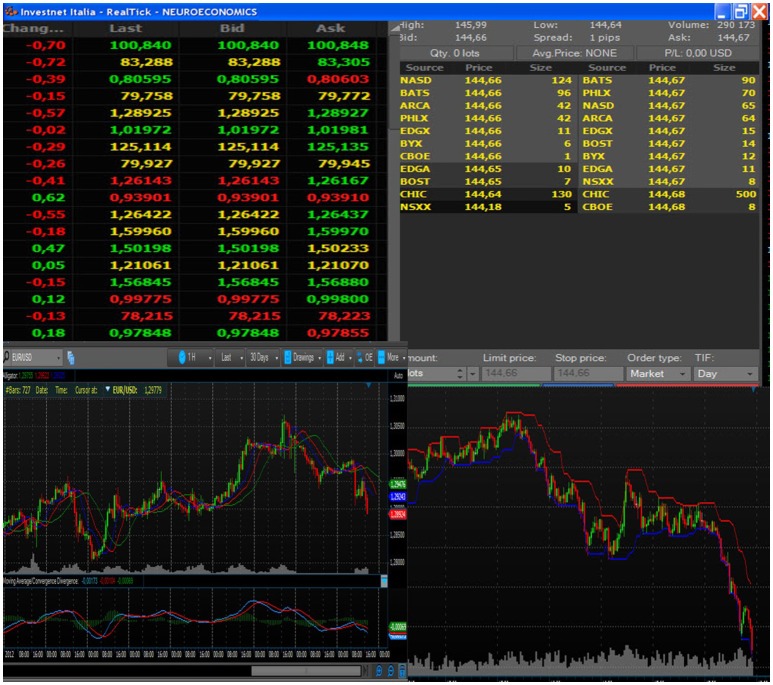
Example of the book of information, composed of both tabular and graphical data. All subjects looked at the chosen stock price and market trends, displayed through this format.

By wearing visors, connected through optic fiber with his laptop placed outside the scanner room, each participant could look at the trading book displayed on the pc screen (Figure 1, C). Independent of the trading platform, professional traders rely on different ways of displaying the same basic information during their activity. These ways consist of figures (often red or green highlighted, depending on the trend), or of graphs. Graphs can display lines or box-and-whiskers or histograms. Each trader usually builds his book of information by adding on the screen multiple tabular or graphical versions of the same stock price trends. In order to reduce variability due to book composition, we agreed with the traders of the team that all the participants would have faced the same kind of information. Figure 2 displays the prototype of this book composition. The trader could negotiate in the market using a special *Track-ball* (Figure 1, T), connected with the laptop (Figure 1, T-C) located outside the fMRI room. The computer input device allowed to control the pointer on the display screen and to insert the decisions of opening and closing positions, reducing movements to a minimum level. In this way, each trader could login to his favorite DAT platform and link the trading outcome to his own bank account. To reduce the number of variables, the participants were requested to negotiate only one financial asset. Every occurred gain, or loss, was recorded immediately in the personal banking account (Figure 1, A–D) and shown on the screen. A continuous supervision of the participant's activity was ensured by a professional trader, staying outside the fMRI room (Figure 1, B). The expert in the control room had the responsibility of controlling the trading activity of the experimental subject, in order to help us understand whether the Buy/Sell proposals actually reflected a professional behavior or just expressed the subjects' willingness of “playing the game.” The expert never intervened during the fMRI sessions; in two cases he alerted us concerning the poor performance of the experimental subjects, and we excluded them from the final data analysis. All participants performed the fMRI session within a 30-day period (mid-November to mid-December) to minimize the influence played by stock market volatility on their behavior. A dedicated system allowed recording the traders' activity synchronously with BOLD signal recording, so as to characterize decision events (recorded through response on the trackball) with reference to their financial meaning, as follows:

- Name of the stock negotiated;
- Type of financial decision event: (i) Proposal to buy (*Ask*); (ii) Proposal to sell (*Bid*), (iii) Updating of proposals (to buy or to sell); (iv) Deletion of proposals (to buy or to sell);
- Value committed in each proposal;
- Stock market price of shares traded;
- Quantity of shares traded in each operation;
- Time (minute and seconds) of any registered decision event;
- Total value of each transaction executed (filled).

Thanks to the collaboration with the IT Service in Milan, we managed to record the activity of the experimental subjects at the same time when the fMRI sessions were carried out and obtain all information corresponding to the trading events in an Excel format. These data gave us the opportunity to measure: the time interval between consecutive operations; the number of the operations carried out during each trading session and the relative fraction of each different type of operation; the average and the total value of the performed transactions.

Each trader underwent one fMRI session lasting 45 min (40 min of functional scanning and five of functional rest for the basal brain activity recording).

### fMRI data acquisition

Anatomical T1-weighted and functional T2^*^-weighted MR images were acquired with a 3 Tesla General Electric scanner equipped with an 8-channel receiver head-coil. Functional images were acquired using a T2^*^-weighted gradient-echo, echo-planar (EPI) pulse sequence (acceleration factor asset 2, 40 sequential transverse slices covering the whole brain, with a TR time of 3,000 ms, TE = 30 ms, flip-angle = 90 degrees, FOV = 205 × 205 mm^2^, inter-slice gap = 0.5 mm, slice thickness = 3 mm, in-plane resolution 2.5 × 2.5 × 2.5 mm^3^). At the beginning of the functional run/session a T1-weighted anatomical scan (acceleration factor arc 2, 156 sagittal slices, matrix 256 × 256, isotropic resolution 1 × 1 × 1 mm^3^, TI = 450 ms, TR = 8,100 ms, TE = 3.2 ms, flip angle 12°) was acquired for each participant.

### Statistical analysis

Functional magnetic imaging data analysis was performed with SPM8 (Statistical Parametric Mapping software; The Wellcome Department of Imaging Neuroscience, London, UK; http://www.fil.ion.ucl.ac.uk) running on MATLAB R2009b (The Mathworks, Inc., Natick, MA). The first four volumes of each run were discarded to allow for T1 equilibration effects. For each participant, all volumes were spatially realigned to the first volume of the first session and un-warped to correct for between-scan motion, and a mean image from the realigned volumes was created. The mean image was then segmented into gray, white, and cerebrospinal fluid and spatially normalized to the Montreal Neurological Institute (MNI) (Evans et al., 1993; Collins et al., 1994). The derived spatial transformation by T1 normalization was applied to the realigned EPIs volumes, which after normalization were re-sampled in 2 × 2 × 2 mm^3^ voxels using trilinear interpolation in space. All functional volumes were then spatially smoothed with a 6-mm full-width half-maximum isotropic Gaussian kernel for the group analysis.

Data were analyzed using a random-effects model (Friston et al., 1999a,b), implemented in a two-level procedure. In the first level, single-subject fMRI responses were modeled in a General Linear Model (GLM) by a design-matrix comprising the onsets and duration of each epoch as follows:

1. The first regressor, named *Response*, refers to the traders' motor response on the trackball in association with their trading decision as described above. The motor response was modeled as one single event lasting 0 s.
2. The second regressor, named *Process*, took into account the epoch occurring 4 s before the Response, containing the moments during which the trader elaborated his decision and, more specifically: the proposal to buy (*Ask*); Proposal to sell (*Bid*), Updating of proposals (to buy or to sell); Deletion of proposals (to buy or to sell);
3. The third regressor refers to the 5 min resting state (explicit baseline);
4. The fourth regressor, the “*regressor of non-interest*,” accounted for the subject's brain activity in all the moments excluded from the three regressors listed above.

In the second level analysis (group-analysis), corresponding contrast images from the first level for each participant were entered into a flexible ANOVA with sphericity-correction for repeated measures (Friston et al., 2002). This model considered the pattern of activation obtained during the mini-epoch named *Process* vs. rest. This model was also used for signal change extraction at the subject level, as specified in the ROI analysis below.

Results were thresholded at *P* < 0.05 family wise error (FWE) corrected at the cluster or voxel level as appropriate (cluster size estimated with a voxel-level threshold of P-uncorrected = 0.005). The location of foci of activation is presented in the stereotaxic space of the MNI coordinate system. Activations were also localized with reference to cytoarchitectonical probabilistic maps of the human brain, using the SPM-Anatomy toolbox v1.7 (Eickhoff et al., 2005).

### ROI analysis

To explore activations as a function of the demographic and professional variables associated with our sample subjects, as well as a function of the traders' behavior during the online trading activity, we further plotted the signal change in specific regions of interest (ROIs). More specifically, 3 ROIs were defined within the functional maps associated with the main effects of *Process* vs. rest. These ROIs reflected the cluster of activation in the ventrolateral prefrontal cortex (vlPFC; 46 50 12), dorsolateral prefrontal cortex (dlPFC; 38 44 38), and right inferior parietal lobule (42 –56 46). Additionally, since the regressor *Process* contained several operations whose merge could have overshadowed interesting modulating effects in emotion-related areas often found in decision making tasks, we selected *post-hoc*—based on literature—two regions in this respect: The nucleus accumbens (NAcc; –12 13 –8; e.g., Sugam et al., 2014; Zalocusky et al., 2016) and the caudate nucleus (NC; –10 2 14; for review, see Balleine et al., 2007). Cortical ROIs were created with a sphere radius of 5 mm, which best represented the extension of activated voxels.

Signal change for each participant was extracted using REX (http://web.mit.edu/swg/rex).

After creating the ROIs, a univariate analysis was carried out to compare the changes in BOLD signal within each ROI with each of the following parameters: age, experience, traders' profiles (risk seeker or risk averse), mean economic value of trader's transactions; economic value of total transactions, distinguishing between buy and sell operations, time intervals between two consecutive decisions, percentage of filled transactions over the total transaction number. Statistical significance was set at *p* ≤ 0.05.

## Results

### Behavioral results associated with the trading activity

A minimum of 20 decision events for each trader was recorded (mean = 45.1± 35.9; range = 20–140). The mean value for each closed (filled) transaction ranged from 185 Euros to 63,000 Euros; (mean = 9590.3 ± 10079.1 Euros). The time interval between two subsequent decision events ranged from 5 to 200 s (mean = 72.7 ± 62.4 s).

### Questionnaire

In order to characterize the trading attitude, the answers collected through the questionnaire underwent factor analysis and distinguished two subgroups of traders. The risk seekers, that we called the *Bold*, were 8/20 cases: they use heuristics, are driven by intuitions and temporary impulses, declare to like the thrill associated with operative decisions of opening, or closing, a personal position. The risk averse, that we called the *Wise*, were 12/20 cases: they declared to stick to the rules and to get the greatest reward from the information processing and computational phases.

No statistically significant differences were found between the two profiles (*Bold* or *Wise*) with respect to variables such as age, experience, commitment. On the other hand, the *Wise* performed fewer financial transactions close to significance (24.8 ± 24.6) with respect to the *Bold* (53 ± 45; *p* = 0.08), with a significantly lower average economic value (5,091 ± 7,200 Euros vs. 14,750 ± 12,953; *p* = 0.05), but with a higher rate of filled proposals (40 vs. 27%).

### fMRI results

**Figure 3:**
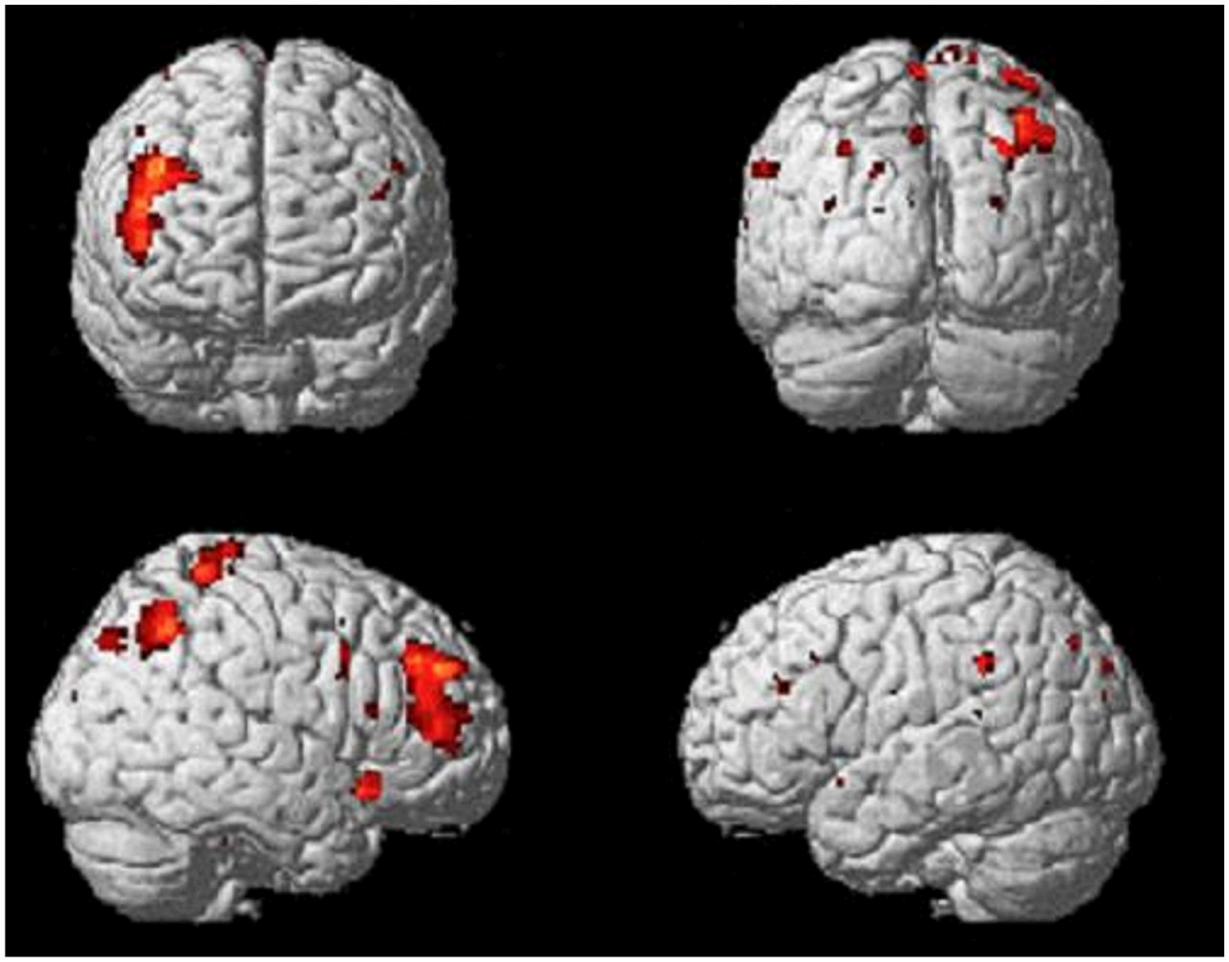
Activations of right lateral prefrontal cortex (LPFC) and right posterior parietal cortex (PPC) from the contrast Process (4 s prior response) vs. rest. Group-averaged statistical parametric maps are rendered onto the MNI brain template (P_FDRcorr_ < 0.05).

Comparing the brain activity 4 s immediately preceding the decisional events with rest (*Process*), results showed an extended enhanced activation of the right lateral prefrontal cortex (maxima: 44 40 36; K_E_ = 589; P_FDR−corr_ = 0.003), and of the posterior parietal cortex, including the inferior parietal lobule (BA40; maxima: 42 –56 46; K_E_ = 301; P_FDR−corr_ = 0.048; Figure 3).

### ROI analysis

ROI analyses were carried out to explore brain activations as a function of the participants' demographic and professional variables, as well as of the traders' behavior during the online trading activity. Table 1 reports the descriptive statistics of BOLD signal changes within each region of interest.

**Table 1:** Average BOLD signal changes in the regions of interest.

	***Mean (SD)***	**Range (min-max)**
Right vlPFC	2.421 (2.473)	–1.22; 6.95
Right dlPFC	2.225 (1.91)	–1.04; 5.70
Right parietal area	2.23 (2.10)	–1.72; 7.33
Left Nucleus Accumbens	0.54 (1.92)	–2.73; 4.99
Left Caudate Nucleus	1.47 (1.51)	–1.27; 3.75

The results of the univariate analysis comparing BOLD signal changes within the regions of interest (ROIs; see section Statistical Analysis above) to personal and professional variables are described in Table 2.

**Table 2:** Results of univariate analysis highlighting the strength of associations between Bold signal changes within ROIs and some personal and professional variables.

**Personal and professional variables**	**BOLD signal changes within the regions of interest**				
	**Right vlPFC**	**Right dlPFC**	**Right parietal area**	**Left nucleus accumbens**	**Left caudate nucleus**
Age	−2.1^*^			−3.2^**^	
Professional age	−2.2^*^				
Traders' profile (Bold vs. Wise)		8.0^**^			13.8^***^
Transaction value of Ask proposal			2.1^*^		
Transaction value of Bid proposal			−2.6^**^		
Time interval between subsequent decision events	2.2^*^	2.8^**^			
Percentage of filled proposals	2.1^*^	2.3^*^			

* p < 0.05;

** p < 0.01;

*** *p < 0.001*.

**Figure 4:**
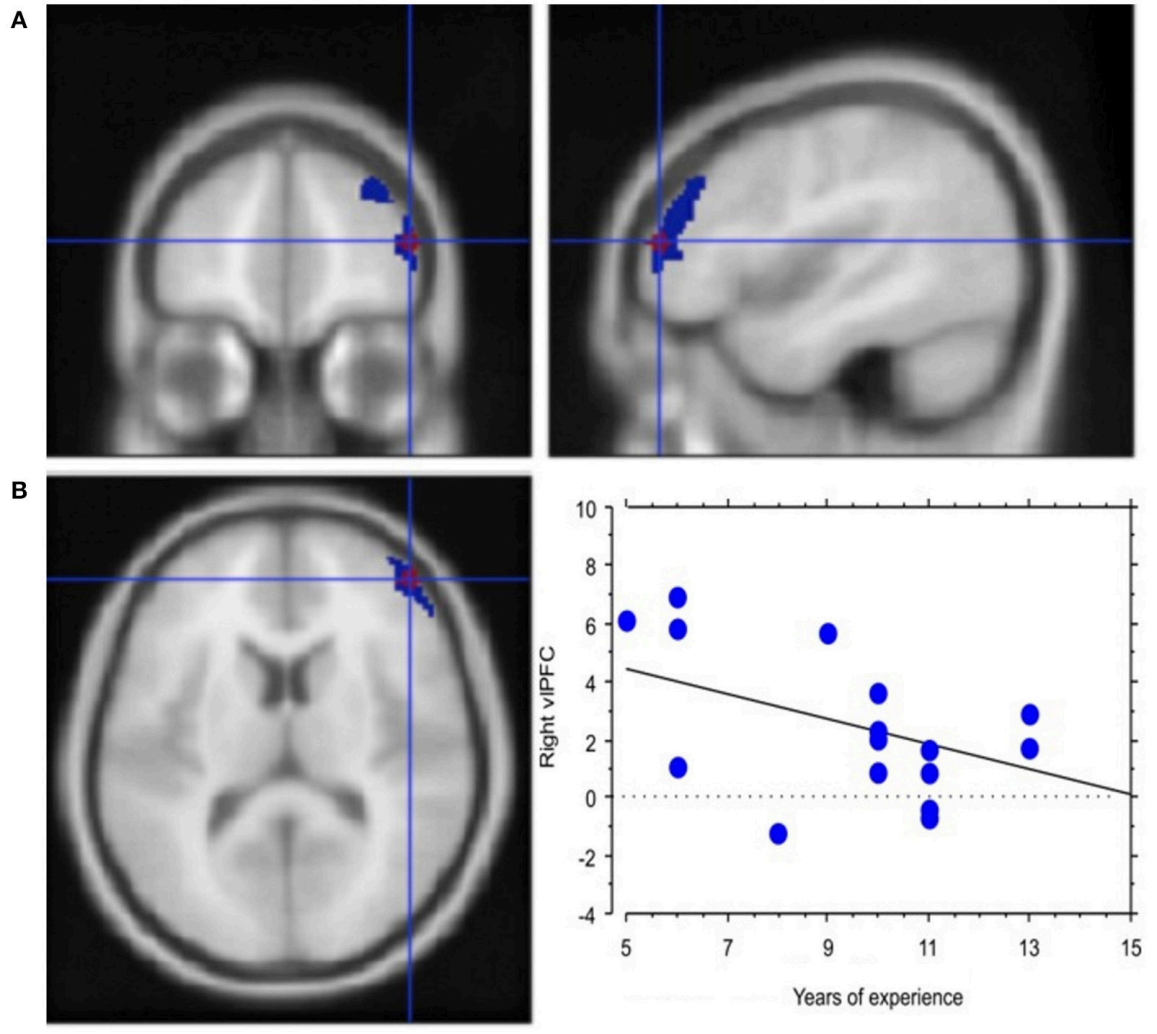
Neural efficiency with experience. **(A)** Activation of right lateral prefrontal cortex (blue) in the contrast *Process* (4 s prior response) vs. rest. In red is the ROI built within the ventrolateral prefrontal activation. Group-averaged statistical parametric maps are rendered onto the MNI brain template (P_FDRcorr_ < 0.05). **(B)** The graph shows the regression between experience and right vlPFC activation in the pre-decisional phase, representing neural efficiency with trading experience (years). Each dot represents a subject.

**Figure 5:**
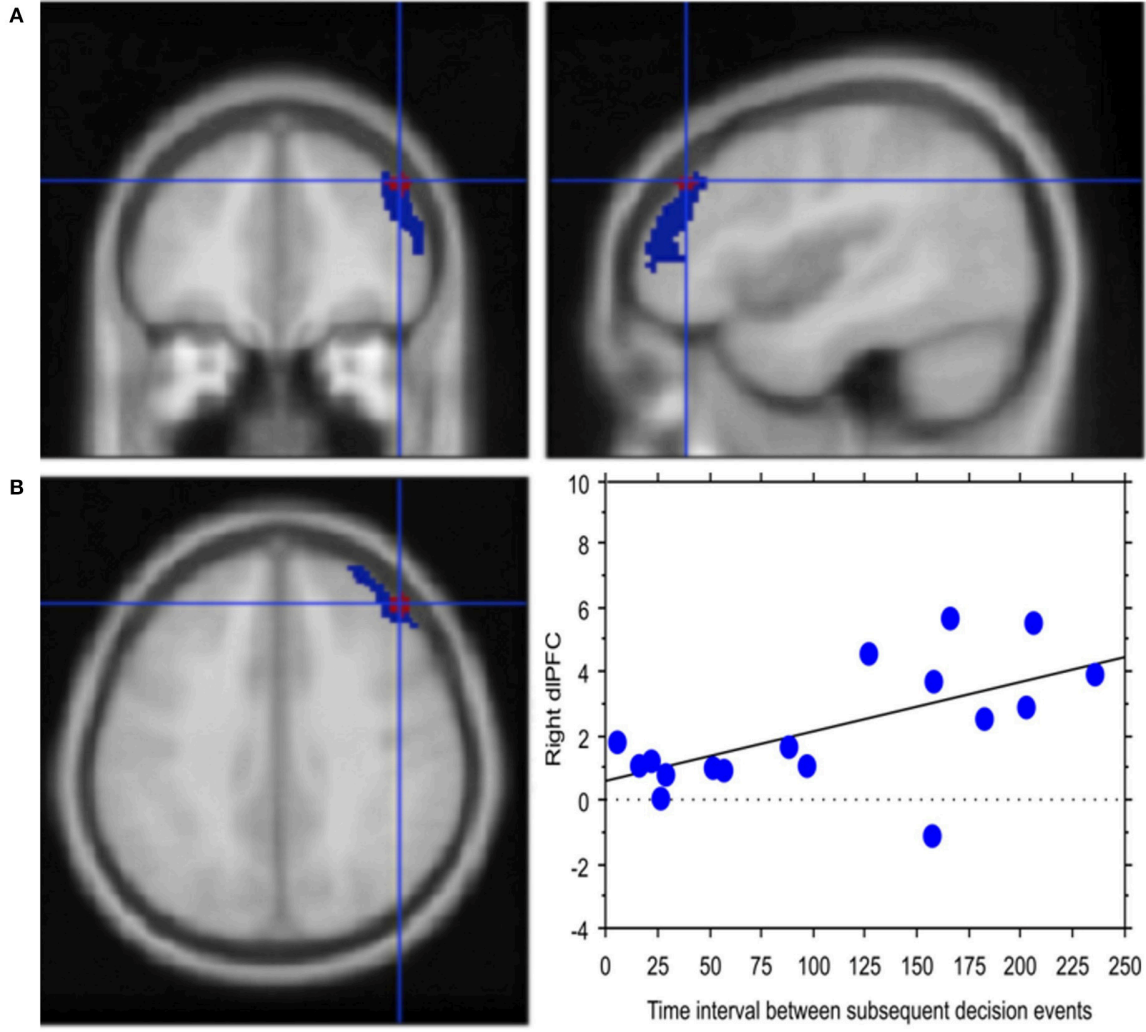
Decision latency is associated with greater cognitive control. **(A)** Activation of right lateral prefrontal cortex (blue) in the contrast *Process* (4 s prior response) vs. rest. In red is the ROI built within the dorsolateral prefrontal activation. Activations are rendered onto the MNI brain template (P_FDRcorr_ < 0.05). **(B)** The graph shows the correlation between right dlPFC activation and decisional latency, representing decision latency in association with greater cognitive control. Each dot represents a subject.

*Experience*, i.e., professional age, mainly modulated vlPFC activity (Y = 6.644 −0.435 ^*^ X; *R*^2^ = 0.239; Figure 4). Additionally, faster decisional activity, with a high number of transactions in short time intervals, was associated with greater activation of the dlPFC (Y = 0.566 +0.015 ^*^ X; *R*^2^ = 0.387; Figure 5). The success of the trading activity, based on a large number of filled transactions was related with higher activation of both vlPFC and dlPFC.

**Figure 6:**
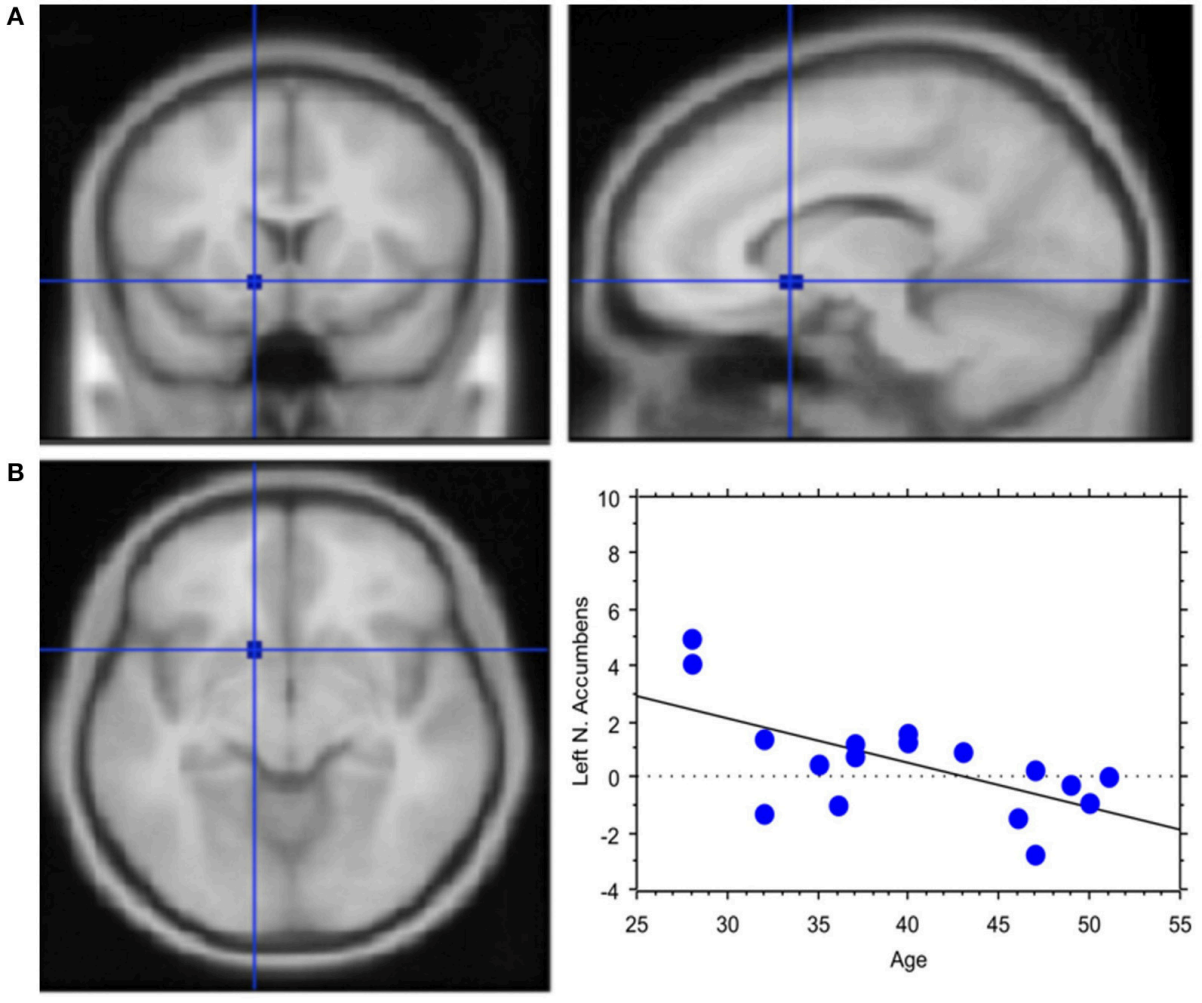
Less emotional involvement with age. **(A)** Region of interest created within left Nucleus Accumbens. Activation in the contrast *Process* (4 s prior response) vs. rest. The ROI is rendered onto the MNI brain template. **(B)** The graph shows the regression between age and left NAcc activation in the pre-decisional phase, which highlights less emotional involvement with the traders' chronological age. Each dot represents a subject.

The total amount of each transaction was related to the BOLD signal change in the posterior Parietal cortex: high value transactions were associated with a stronger activation of this area when subjects' buy rather than sell. Moreover, an inverse correlation was found between age and signal changes in left NAcc (Y = 7.002–0.162 ^*^ X; *R*^2^ = 0.408; Figure 6).

**Figure 7:**
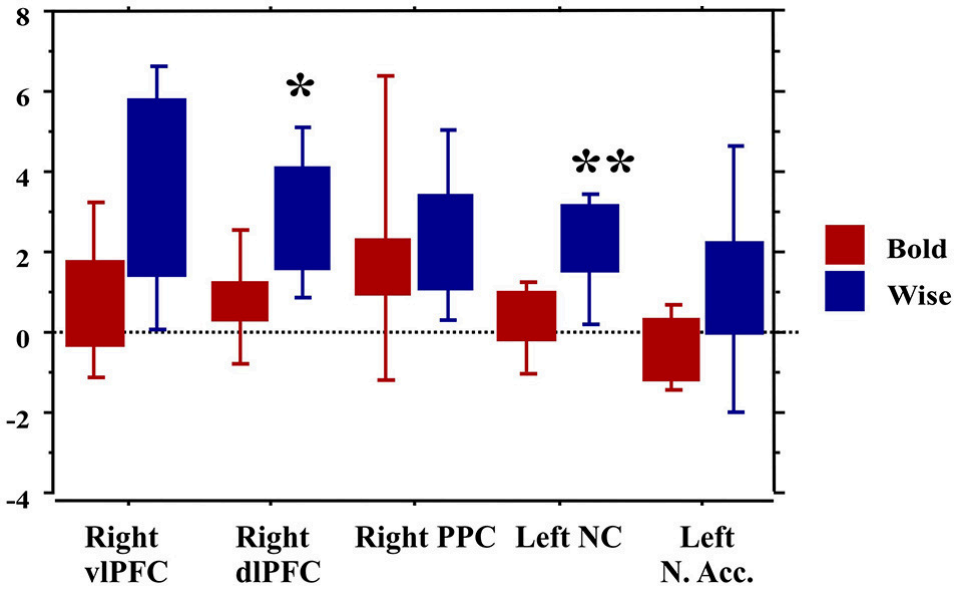
Brain activity in the regions of interest as a function of the traders' profile. ^*^p < 0.01; ^**^p < 0.001.

Finally, the traders' profile also proved to be an important factor modulating brain activity. Compared to the Bold, the Wise subjects showed significantly greater activations of areas involved in value processing (Left caudate nucleus) and selection of final decision (Right dlPFC), this finding being independent of age and professional expertise (Figure 7).

## Discussion

This research represents the first attempt, in the current neuroeconomics literature, to detect the neural aspects of financial decision making through studying professional traders involved in real stock market negotiations using their own real money.

The recurrent and ruinous financial bubbles, monetary crisis and stock market crashes prove a serious weakness of classic economic and financial theories and an evident failure of sophisticated models to describe investors' expected behavior. Markowitz's *Modern Portfolio Theory* (Markowitz, 1952) posits that risk can be estimated by the variability of the expected reward (variance), regardless of the context, while, on the other hand, reward consists only in stocks' returns. Reality is more complex. Risk is a complicated (and variable) mix between the probabilities of occurrence of a future event and the economic value of the effect (positive or negative) due to its materialization.

It is difficult to estimate the probabilities and economic values of a future event. Difficulties increase if we consider emotional factors, personal risk preferences, attitude to speculative risk, personal experiences, culture, mental status, and so on (Schiebener and Brand, 2015). Moreover, reward cannot be always associated to monetary gains. Classical visions, in fact, fail to take into account the role played by emotions.

With the present study we introduced an original investigation protocol aimed at unveiling to what extent individual and context variables affect decisions of professional traders involved in real asset negotiations. The novelty of the research mainly consists in the arrangement of a real market setting; in fact, differently from current scientific investigations, we exposed our participants to their usual trading activity, allowing them to refer to their preferred trading platform, and weight pros and cons of buying/selling assets using their own bank account. The decision of pursuing such a complex goal was made in order to overcome the limitations related to the ecological validity of results obtained through fMRI protocols so far applied to the study of financial decisions. In the past, many researchers have questioned whether economic decision making can truly be measured and generalized in such a restricted situation as that tested in the fMRI-scanner, where only “fictive” tasks and not “real-world” situations can be studied (Ariely and Berns, 2010; Ayaz et al., 2013).

In this study, we succeeded in connecting the real market to the fMRI scanner, thus providing the experimental subjects with stimuli that were similar to those experienced in their routine activity. We introduced several control measures/strategies in order to reduce the huge variability expected in such a complex investigation setting. Namely, the trading book was standardized in order to reduce variability due to different color, shape and format of information concerning the asset price; trading was allowed for one preferred asset per subject, and only on the Italian market, considering prices' trend in a limited time period (for 1 month). Finally, the whole trading activity carried out by each subject during the fMRI session was recorded and judged by expert traders in the offline condition, in order to exclude those subjects who just “played the game,” i.e., pretended to negotiate while limiting themselves to put forward their offer and immediately canceling them without really buying or selling any assets. Moreover, the continuous recording of the real market trends during the fMRI session allowed us to synchronize the information flow with the decisional events (as indicated by the trader's response on the trackball) and with BOLD signal.

During the time period that we chose to analyse, i.e., 4 s preceding the decisional event, the participants were expected to evaluate sensory input (market trend) based on prior knowledge and experiences made in similar situations, and to form an anticipation of the potential outcome on the basis of probabilities of expected outcomes and the magnitude of the reward in conjunction with previous experiences (Ernst et al., 2004). Our results showed a strong activation of cortical areas lateralized to the right hemisphere when comparing BOLD signal during the decisional phase (4 s prior motor response) with baseline. Cortical activations involved the posterior parietal area, including the intraparietal lobule (IPL) and a large part of the lateral prefrontal cortex (dorsal and ventral). These cortical areas, playing different roles, are notably recognized in decision making processes.

Our experiences allow us to determine the predictive value of our choices. Value representation strongly involves the role of the orbitofrontal cortex, whereas the translation of value attribution in a behavioral response pertains to the functional role of the parietal cortex and, particularly, the intraparietal lobule (IPL). More specifically, values modulate decisions in the parietal cortex, which associates sensory information with motor commands, guiding attention. Sensory-motor processes and attention therefore, incorporate the value of alternative interpretations of sensory signals that are used to guide behavior (see, for example, Cohen and Andersen, 2002; Padoa-Schioppa and Assad, 2006; Glimcher and Fehr, 2013). IPL, therefore, associates sensation and potential behavior according to the expected value of a series of possible behavioral responses (Hansen et al., 2012). Additionally, activity in IPL is associated to the memory of past results. Single neurons response in IPL to a specific target corresponds to the history of the relative gains associated with that target. A greater weight is given to the most recent events (Yang and Shadlen, 2007). Importantly, its activity is independent of “what” (food, sex, social factors, etc.) and modality—“how” (visual, tactile, olfactory, etc.; Deaner et al., 2005; Hayden et al., 2007; Klein et al., 2008). Afferent signals to IPL have been already converted in a fungible value in other areas (e.g., the orbitofrontal cortex; see for example Padoa-Schioppa and Assad, 2006; Padoa-Schioppa, 2007). This description of the functional role of IPL in decision making is in line with our results showing right IPL activation in the period right preceding the actual decision-related motor response, and namely when the traders processed their decision. It is not surprising that our parietal activation was not affected by factors associated with either the participants' demographic and professional variables, or by the traders' behavioral variable during the online trading activity (see section Methods above). IPL is deputed to compute decisions based on expected values built upon experience and to associate that decision to a motor command, a process that, in our study, was emphasized by the trader's ongoing decisional process.

Other studies have reported a similar activity to that observed in IPL in areas, including the prefrontal cortex, caudate nucleus, substantia nigra pars reticulata, and superior colliculus. Also in these area the neural activity appears to be correlated either to the probability of movement in a given direction or to the marginal magnitude of the expected reward (Salzman et al., 2005; Hikosaka et al., 2006). In the present study we observed a large activation of right lateral prefrontal cortex, which is generally associated with probability estimation of different (relative) values assigned to the expected reward for each possible choice (Platt and Glimcher, 1999; Poldrack et al., 1999; Weickert et al., 2009; d'Acremont et al., 2013; Bisbing et al., 2015). The lateral prefrontal area is generally involved in cognitive control that allows information processing and behavior to vary adaptively from moment to moment depending on current goals. To better outline the functional role of the large PFC activation found in the present study, it was subdivided in two sectors: a dorsal and ventral one. With respect to the dorsal part of lateral PFC, its primary role is most likely the selection of information and preparation for motor execution (Pochon et al., 2001; Passingham and Sakai, 2004; Sakai, 2008), as well as potentially the monitoring and the early upregulation of information processing in task-related sensory-motor regions, or task networks (Kelly and Garavan, 2005; Halsband and Lange, 2006). Shallice (2004) suggested that the right dlPFC is primarily involved in monitoring if a configured motor plan is executed in accordance with the task goals (see also Vogt et al., 2007). Its activation in the present study then plausibly reflects monitoring of and attention to ongoing operations. The “attentional load” would then represent the cognitive load associated with each trader's decisional process. Additionally, a recent meta-analysis (Cieslik et al., 2013) has shown that cognitive action control in the right dlPFC relies on differentiable neural networks and cognitive functions. In particular, the posterior subregion shows increased connectivity with bilateral intraparietal sulci, whereas the anterior subregion shows increased connectivity with the anterior cingulate cortex. Functional characterization with quantitative forward and reverse inferences revealed that, whereas the anterior network is more strongly associated with attention and action inhibition processes, the posterior network is more strongly related to action execution and working memory.

Congruent with this idea, regression analysis carried out in our study relating BOLD signal in dlPFC with the traders' response latency, i.e., the interval between subsequent decisional events, highlighted a positive correlation between signal change and decision latency. This finding supports the relationship between decision latency and sustained monitoring, which would then translate into greater cognitive control (Petersen et al., 1998; Shallice, 2004), which typically reduces with practice (Kelly and Garavan, 2005; Dayan and Cohen, 2011).

Interestingly, this practice effect was observed in the vlPFC activation profile. In fact, our regression results showed a negative correlation between activation of vlPFC and years of trading experience, which can be easily associated with the concept of *neural efficiency*. Neural efficiency, in fact, shows in the decrease, extent and intensity of activations in cognitive control structures (see Babiloni et al., 2009, 2010). The ability to apply behavioral strategies to obtain rewards *efficiently* and make choices based on changes in the value of rewards is fundamental to the adaptive control of behavior, a quality evidently needed by our expert traders during their online and continuous activity. Rhesus monkeys with bilateral ablations of the vlPFC on tasks that required the use of behavioral strategies to optimize the rate with which rewards were accumulated were specifically impaired in performing the strategy-based task, but not value-based decision-making, which—on the other hand—is processed in the orbitofrontal region (Baxter et al., 2009). This functional description of vlPFC highlights the role of vlPFC in strategic planning that, alongside the neural efficiency theory, remains in line with our results in that traders with longer experience are possibly equipped with pre-established strategic options and, hence, with heuristics that allow them to efficiently respond with a low activation cost (for a detailed meta-analysis of vlPMC, see Levy and Wagner, 2011).

In this research we did not dissociate the different component phases of decision-making. Therefore, the observed neural activity refers both to the selection phase during which information concerning price trends was assessed and valued, as well as reward anticipation (Ernst et al., 2004). Consequently, signal change recorded in the decisional period contained several operations whose merge could have overshadowed interesting modulating effects in emotion-related areas often found in decision-making tasks. In this view, based on literature, two further regions of interest were created to evaluate the possible modulating effect of our trader's profile on emotion-related structures: the caudate nucleus (for review, see Balleine et al., 2007)—part of the dorsal striatum—and the nucleus accumbens (e.g., Sugam et al., 2014; Zalocusky et al., 2016)—part of the ventral striatum. These areas, alongside the anterior insula, the anterior cingulate cortex, posterior cingulate cortex, and orbitofrontal cortex, are part of the choice evaluation network (Preuschoff et al., 2006). Monetary reward anticipation typically involves activation of ventral striatum and the most anterior portion of the orbitofrontal cortex (Knutson et al., 2005; Sescousse et al., 2013). On the other hand, the dorsal striatum has long been acknowledged as an integral component of the reward circuitry responsible for the control of motivated behavior (Delgado et al., 2003). Using event-related fMRI, it was found that dorsal striatum activity is more robust during the early phases of learning, i.e., initial stages of trial and error learning, whereas its signal decreases as learning progresses, suggesting an evolving adaptation of reward feedback expectancy as the behavior-outcome contingency becomes more predictable (Delgado et al., 2005).

Activation of ventral striatum in our study was negatively associated with the trader's chronological age, suggesting less emotional involvement with age. This finding confirms previous observations. In fact, in 316 healthy adults aged 20–89 years, involved in a judgment task, middle-age subjects were characterized by decreased modulation to task-demand in subcortical regions (nucleus caudate, nucleus accumbens, thalamus), whereas very old individuals showed reduced modulation to task difficulty in midbrain/brainstem regions (ventral tegmental, substantia nigra; Kennedy et al., 2015). Hence, it has been hypothesized that activation to cognitive demand lowers following a gradient along the dopaminergic/nigrostriatal system, with an early manifestation of deficits in subcortical nuclei in middle-age and then to midbrain/brainstem dopaminergic regions in the very old.

A further interesting result was found when comparing activation in our ROIs as a function of the participants' trading activity. In this respect, they were divided into those who matched what we named a Bold and a Wise attitude to the trading activity. The Bold—or risk seekers—declared to like the thrill associated with operative decisions of opening, or closing, a personal position. The Wise—risk averse—declared to stick to the rules and to get the greatest reward from the information processing and computational phases. Our regression analysis showed a significantly lower activation of the dorsal striatum for the Bold compared to the Wise.

According to mainstream theories, risky decision making assume that the core processes involved in reaching a risky decision include weighting each payoff or reward magnitude by its probability and then summing the expected outcomes (negative or positive; Rao et al., 2012). In the so-called payoff network, the striatum is the structure most consistently reported to be involved in the influence of reward magnitude/payoff on the neural substrate. Although, many neuroimaging studies of reward processes have focused on ventral striatal activation, a growing body of literature from animal and human studies suggests that the dorsal striatum is also involved in motivated behaviors. While ventral striatum activity is thought to be associated with the anticipation of larger rewards, the dorsal striatum is expected to activate when both rewards and punishments of larger magnitude are anticipated (Knutson et al., 2001). Since neural activation in the ventromedial caudate nucleus during anticipation of both gain and loss has been found to be decreased in patients with pathological gambling, compared with that in healthy controls (Balodis et al., 2012; Choi et al., 2012) the observation of a reduced activity in this area in the self-judged Bold traders, in a real-trading scenario, could be interpreted as a potential marker of greater susceptibility of these subjects to an addictive, more than reflexive, behavior. This interpretation is in line with the idea of a poor engagement of the dlPFC for cognitive control.

Notwithstanding the fact that an ecological setting may be regarded as a limitation of the study, due to the challenge of facing countless independent variables, when we compared our data to the existing literature on decision making, we observed the activation of the same networks expected to be engaged during speculative risky choices, i.e., a probability and a payoff network: the former included the right lateral prefrontal cortex and posterior parietal cortex, while the latter included the left ventral and dorsal striatum. Moreover, brain activity in these areas, during the pre-decisional phase, proved to be modulated by factors like age and experience in agreement with previous reports, while a greater cognitive control, expressed by the involvement of the dlPFC, correlated with a longer decision time and a greater decision efficiency.

Finally, different attitudes to direct access trading, as perceived and witnessed by the traders involved in our experiment, correlated with distinct brain activity profiles: in particular, a lower or absent activation of both the caudate nucleus and the dlPFC characterized those subjects who described themselves as very self-confident, managing trading with a certain amount of recklessness. It is conceivable that these subjects handle trading as a well-learned automatic behavior, largely relying on heuristics thus reducing to a minimum their cognitive effort. This approach does not necessarily turns out into a success: in fact, in the so-called *Bold* subjects, an intense decision activity corresponds to a lower rate of finalized transactions, than that obtained by the more reflexive *Wise* traders.

These preliminary results should prompt an update of the classic economic vision: it is not acceptable to assume that financial agents are always well informed, not emotionally-dependent, conscious and rational and able to estimate perfectly probabilities and economic value of any risk faced. The trader who can confidentially evaluate risk via statistical models without any influence of emotion or intuition does not exist. These neuro-economic findings suggest the need to conduct other neuroeconomics studies to improve traditional asset pricing models and investors' behavior theories, to take into account the role of emotions and unconscious processes. Further research developments should investigate which neural circuits handle financial decision making and which anticipate the final choice adopted; eventually, real-life scenarios should become a relevant component of the experimental protocols adopted.

## Author contributions

GR and MC equally contributed to the Conception and design of the work; CD and MC contributed to Data collection and data analysis and interpretation; LF, CD, and MC prepared the draft article, that was critically revised by GR and finally approved by all the authors

### Conflict of interest statement

The authors declare that the research was conducted in the absence of any commercial or financial relationships that could be construed as a potential conflict of interest.

## References

[B1] Ariely D. Berns G. S. (2010). Neuromarketing: the hope and hype of neuroimaging in business. Nat. Rev. Neurosci. 11, 284–292. 10.1038/nrn2795 20197790 PMC2875927

[B2] Ayaz H. Onaral B. Izzetoglu K. Shewokis P. A. McKendrick R. Parasuraman R. (2013). Continuous monitoring of brain dynamics with functional near infrared spectroscopy as a tool for neuroergonomic research: empirical examples and a technological development. Front. Hum. Neurosci. 7:871. 10.3389/fnhum.2013.00871 24385959 PMC3866520

[B3] Babiloni C. Frisoni G. B. Del Percio C. Zanetti O. Bonomini C. Cassetta E.. (2009). Ibuprofen treatment modifies cortical sources of EEG rhythms in mild Alzheimer's disease. Clin. Neurophysiol. 120, 709–718. 10.1016/j.clinph.2009.02.005 19324592

[B4] Babiloni C. Marzano N. Infarinato F. Iacoboni M. Rizza G. Aschieri P.. (2010). “Neural efficiency” of experts' brain during judgment of actions: a high-resolution EEG study in elite and amateur karate athletes. Behav. Brain Res. 207, 466–475. 10.1016/j.bbr.2009.10.034 19891991

[B5] Bach D. R. Dolan R. J. (2012). Knowing how much you don't know: a neural organization of uncertainty estimates. Nat. Rev. Neurosci. 13, 572–586. 10.1038/nrn3289 22781958

[B6] Balleine B. W. Delgado M. R. Hikosaka O. (2007). The role of the dorsal striatum in reward and decision-making. J. Neurosci. 27, 8161–8165. 10.1523/JNEUROSCI.1554-07.2007 17670959 PMC6673072

[B7] Balodis I. M. Kober H. Worhunsky P. D. Stevens M. C. Pearlson G. D. Potenza M. N. (2012). Diminished frontostriatal activity during processing of monetary rewards and losses in pathological gambling. Biol. Psychiatry 71, 749–757. 10.1016/j.biopsych.2012.01.006 22336565 PMC3460522

[B8] Barber B. M. Odean T. (2000). Trading is hazardous to your wealth: the common stock investment performance of individual investors. J. Finance 55, 773–806. 10.1111/0022-1082.00226

[B9] Baxter M. G. Gaffan D. Kyriazis D. A. Mitchell A. S. (2009). Ventrolateral prefrontal cortex is required for performance of a strategy implementation task but not reinforcer devaluation effects in rhesus monkeys. Eur. J. Neurosci. 29, 2049–2059. 10.1111/j.1460-9568.2009.06740.x 19453635 PMC2688497

[B10] Benartzi S. Thaler R. (1995). Myopic loss aversion and the equity premium puzzle. Q. J. Econ. 110, 73–92. 10.2307/2118511

[B11] Bisbing T. A. Olm C. A. McMillan C. T. Rascovsky K. Baehr L. Ternes K.. (2015). Estimating frontal and parietal involvement in cognitive estimation: a study of focal neurodegenerative diseases. Front. Hum. Neurosci. 9:317. 10.3389/fnhum.2015.00317 26089786 PMC4454843

[B12] Bruguier A. J. Quartz S. R. Bossaerts P. (2010). Exploring the nature of “Trader Intuition”. J. Finance 65, 1703–1723. 10.1111/j.1540-6261.2010.01591.x

[B13] Burke C. J. Tobler P. N. (2011). Coding of reward probability and risk by single neurons in animals. Front. Neurosci. 5:121. 10.3389/fnins.2011.00121 22013410 PMC3190139

[B14] Camerer C. F. Hogarth R. (1999). The effects of financial incentives in experiments: a review and capital-labor-production framework. J. Risk Uncertain. 19, 7–42. 10.1023/A:1007850605129

[B15] Choi J. S. Shin Y. C. Jung W. H. Jang J. H. Kang D. H. Choi C. H.. (2012). Altered brain activity during reward anticipation in pathological gambling and obsessive-compulsive disorder. PLoS ONE 7:e45938. 10.1371/journal.pone.0045938 23029329 PMC3447818

[B16] Cieslik E. C. Zilles K. Caspers S. Roski C. Kellermann T. S. Jakobs O.. (2013). Is there “one” DLPFC in cognitive action control? Evidence for heterogeneity from co-activation-based parcellation. Cereb. Cortex 23, 2677–2689. 10.1093/cercor/bhs256 22918987 PMC3792742

[B17] Clithero J. A. Tankersley D. Huettel S. A. (2008). Foundations of neuroeconomics: from philosophy to practice. PLoS Biol. 6:e298. 10.1371/journal.pbio.0060298 19067493 PMC2586372

[B18] Coates J. Gurnell M. Rustichini A. (2009). Second-to-fourth digit ratio predicts success among high-frequency financial traders. Proc. Natl. Acad. Sci. U.S.A. 106, 623–628. 10.1073/pnas.0810907106 19139402 PMC2626753

[B19] Coates J. M. Herbert J. (2008). Endogenous steroids and financial risk taking on a London trading floor. Proc. Natl. Acad. Sci. U.S.A. 105, 6167–6172. 10.1073/pnas.0704025105 18413617 PMC2329689

[B20] Cohen Y. E. Andersen R. A. (2002). A common reference frame for movement plans in the posterior parietal cortex. Nat. Rev. Neurosci. 3, 553–562. 10.1038/nrn873 12094211

[B21] Collins D. L. Neelin P. Peters T. M. Evans A. C. (1994). Automatic 3D intersubject registration of MR columetric data in standardized Talairach space. J. Comput. Assist. Tomogr. 18, 192–205. 10.1097/00004728-199403000-00005 8126267

[B22] d'Acremont M. Bossaerts P. (2008). Neurobiological studies of risk assessment: a comparison of expected utility and meanvariance approaches. Cogn. Affect. Behav. Neurosci. 8, 363–374. 10.3758/CABN.8.4.363 19033235

[B23] d'Acremont M. Fornari E. Bossaerts P. (2013). Activity in inferior parietal and medial prefrontal cortex signals the accumulation of evidence in a probability learning task. PLoS Comput. Biol. 9:e1002895. 10.1371/journal.pcbi.1002895 23401673 PMC3561043

[B24] Dayan E. Cohen L. G. (2011). Neuroplasticity subserving motor skill learning. Neuron 72, 443–454. 10.1016/j.neuron.2011.10.008 22078504 PMC3217208

[B25] Deaner R. O. Khera A. V. Platt M. L. (2005). Monkeys pay per view: adaptive valuation of social images by rhesus macaques. Curr. Biol. 15, 543–548. 10.1016/j.cub.2005.01.044 15797023

[B26] Delgado M. R. Locke H. M. Stenger V. A. Fiez J. A. (2003). Dorsal striatum responses to reward and punishment: effects of valence and magnitude manipulations. Cogn. Affect. Behav. Neurosci. 3, 27–38. 10.3758/CABN.3.1.27 12822596

[B27] Delgado M. R. Miller M. M. Inati S. Phelps E. A. (2005). An fMRI study of reward-related probability learning. Neuroimage 24, 862–873. 10.1016/j.neuroimage.2004.10.002 15652321

[B28] De Martino B. O'Doherty J. Ray D. Bossaerts P. Camerer C. (2013). In the mind of the market: theory of mind biases value computation during financial bubbles. Neuron 79, 1222–1231. 10.1016/j.neuron.2013.07.003 24050407 PMC3781325

[B29] Eickhoff S. B. Stephan K. E. Mohlberg H. Grefkes C. Fink G. R. Amunts K.. (2005). A new SPM toolbox for combining probabilistic cytoarchitectonic maps and functional imaging data. Neuroimage 25, 1325–1335. 10.1016/j.neuroimage.2004.12.034 15850749

[B30] Ernst M. Nelson E. E. McClure E. B. Monk C. S. Munson S. Eshel N.. (2004). Choice selection and reward anticipation: an fMRI study. Neuropsychologia 42, 1585–1597. 10.1016/j.neuropsychologia.2004.05.011 15327927

[B31] Evans A. C. Collins D. L. Mills S. R. Brown E. D. Kelly R. L. Peters T. M. (1993). 3D statistical neuroanatomical models from 305 MRI volumes, in Proceedings of IEEE-Nuclear Science Symposium and Medical Imaging Conference, Vol. 95 (London: MTP), 1813–1817.

[B32] Friston K. J. Glaser D. E. Henson R. N. Kiebel S. Phillips C. Ashburner J. (2002). Classical and Bayesian inference in neuroimaging: applications. Neuroimage 16, 484–512. 10.1006/nimg.2002.1091 12030833

[B33] Friston K. J. Holmes A. P. Price C. J. Büchel C. Worsley K. J. (1999a). Multisubject fMRI studies and conjunction analyses. Neuroimage 10, 385–396. 10.1006/nimg.1999.0484 10493897

[B34] Friston K. J. Holmes A. P. Worsley K. J. (1999b). How many subjects constitute a study? Neuroimage 10, 1–5. 10.1006/nimg.1999.0439 10385576

[B35] Frydman C. Barberis N. Camerer C. Bossaerts P. Rangel A. (2014). Using neural data to test a theory of investor behavior: an application to realization utility. J. Finance 69, 907–946. 10.1111/jofi.12126 25774065 PMC4357577

[B36] Genevsky A. Knutson B. (2015). Neural affective mechanisms predict market-level microlending. Psychol. Sci. 26, 1411–1422. 10.1177/0956797615588467 26187248 PMC4570982

[B37] Gervais S. Odean T. (2001). Learning to be overconfident. Rev. Financ. Stud. 14, 1–27. 10.1093/rfs/14.1.1

[B38] Glimcher P. W. Fehr E. (eds.). (2013). Neuroeconomics: Decision Making and the Brain. London: Elsevier.

[B39] Gneezy U. Potters J. (1997). An experiment on risk taking and evaluation periods. Q. J. Econ. 112, 631–645. 10.1162/003355397555217

[B40] Grinblatt M. Keloharju M. (2009). Sensation seeking, overconfidence, and trading activity. J. Finance 64, 549–578. 10.1111/j.1540-6261.2009.01443.x

[B41] Halsband U. Lange R. K. (2006). Motor learning in man: a review of functional and clinical studies. J. Physiol. 99, 414–424. 10.1016/j.jphysparis.2006.03.007 16730432

[B42] Hansen K. A. Hillenbrand S. F. Ungerleider L. G. (2012). Human brain activity predicts individual differences in prior knowledge use during decisions. J. Cogn. Neurosci. 24, 1462–1475. 10.1162/jocn_a_00224 22401286 PMC7410366

[B43] Harrison G. W. Rutström E. E. (2008). Experimental evidence on the existence of hypothetical bias in value elicitation methods, in Handbook of Experimental Economics Results eds Plott C. Smith V. (Amsterdam: Elsevier), 752–767. 10.1016/S1574-0722(07)00081-9

[B44] Hayden B. Y. Parikh P. C. Deaner R. O. Platt M. L. (2007). Economic principles motivating social attention in humans. Proc. R. Soc. B 274, 1751–1756. 10.1098/rspb.2007.0368 17490943 PMC2493582

[B45] Heaton J. B. (2002). Managerial optimism and corporate finance. Financ. Manage. 31, 33–45. 10.2307/3666221

[B46] Hensher D. A. (2010). Hypothetical bias, choice experiments and willingness to pay. Transport. Res. Part B 44, 735–752. 10.1016/j.trb.2009.12.012

[B47] Hertwig R. Ortmann A. (2001). Experimental practices in economics: a methodological challenge for psychologists? J. Behav. Brain Sci. 24, 383–403. 11682798 10.1037/e683322011-032

[B48] Hikosaka O. Nakamura K. Nakahara H. (2006). Basal ganglia orient eyes to reward. J. Neurophysiol. 95, 567–584. 10.1152/jn.00458.2005 16424448

[B49] Hirshleifer D. Shumway T. (2003). Good day sunshine: stock returns and the weather. J. Finance 58, 1009–1032. 10.1111/1540-6261.00556

[B50] Huber R. E. Klucharev V. Rieskamp J. (2015). Neural correlates of informational cascades: brain mechanisms of social influence on belief updating. Soc. Cogn. Affect. Neurosci. 10, 589–597. 10.1093/scan/nsu090 24974396 PMC4381243

[B51] Kelly M. C. Garavan H. (2005). Human functional neuroimaging of brain changes associated with practice. Cereb. Cortex 15, 1089–1102. 10.1093/cercor/bhi005 15616134

[B52] Kennedy K. M. Rodrigue K. M. Bischof G. N. Hebrank A. C. Reuter-Lorenz P. A. Park D. C. (2015). Age trajectories of functional activation under conditions of low and high processing demands: an adult lifespan fMRI study of the aging brain. Neuroimage 104, 21–34. 10.1016/j.neuroimage.2014.09.056 25284304 PMC4252495

[B53] Klein J. T. Deaner R. O. Platt M. L. (2008). Neural correlates of social target value in macaque parietal cortex. Curr. Biol. 18, 419–424. 10.1016/j.cub.2008.02.047 18356054 PMC2362498

[B54] Knutson B. Adams C. M. Fong G. W. Hommer D. (2001). Anticipation of increasing monetary reward selectively recruits nucleus accumbens. J. Neurosci. 21:RC159. Available online at: http://www.jneurosci.org/cgi/content/full/5472 11459880 10.1523/JNEUROSCI.21-16-j0002.2001 PMC6763187

[B55] Knutson B. Taylor J. Kaufman M. Peterson R. Glover G. (2005). Distributed neural representation of expected value. J. Neurosci. 25, 4806–4812. 10.1523/JNEUROSCI.0642-05.2005 15888656 PMC6724773

[B56] Kuhnen C. M. Knutson B. (2011). The influence of affect on beliefs, preferences, and financial decisions. J. Financ. Quant. Anal. 46, 605–626. 10.1017/S0022109011000123

[B57] Levy B. J. Wagner A. D. (2011). Cognitive control and right ventrolateral prefrontal cortex: reflexive reorienting, motor inhibition, and action updating. Ann. N.Y. Acad. Sci. 1224, 40–62. 10.1111/j.1749-6632.2011.05958.x 21486295 PMC3079823

[B58] Lima Filho R. I. R. Rocha F. A. Massad E. (2015). Traders decision-making processes: results from an investment simulation monitored with an EEG, in 2015 NeuroPsychoEconomics Conference Proceedings (Washington: JNPE), 11, 1–62.

[B59] Lo A. Repin D. (2002). The psychophysiology of real-time financial risk processing. J. Cogn. Neurosci. 14, 323–339. 10.1162/089892902317361877 11970795

[B60] Malmendier U. Tate G. (2005). CEO overconfidence and corporate investment. J. Finance 60, 2661–2700. 10.1111/j.1540-6261.2005.00813.x

[B61] Markowitz H. M. (1952). Portfolio selection. J. Finance 7, 77–91. 10.1111/j.1540-6261.1952.tb01525.x

[B62] Mohr P. N. Biele G. Heekeren H. R. (2010). Neural processing of risk. J. Neurosci. 30, 6613–6619. 10.1523/JNEUROSCI.0003-10.2010 20463224 PMC6632558

[B63] Odean T. (1998). Are investors reluctant to realize their losses? J. Finance 53, 1775–1798. 10.1111/0022-1082.00072

[B64] Padoa-Schioppa C. (2007). Orbitofrontal cortex and the computation of economic value. Ann. N.Y. Acad. Sci. 1121, 232–253. 10.1196/annals.1401.011 17698992

[B65] Padoa-Schioppa C. Assad J. A. (2006). Neurons in orbitofrontal cortex encode economic value. Nature 441, 223–226. 10.1038/nature04676 16633341 PMC2630027

[B66] Passingham D. Sakai K. (2004). The prefrontal cortex and working memory: physiology and brain imaging. Curr. Opin. Neurobiol. 14, 163–168. 10.1016/j.conb.2004.03.003 15082320

[B67] Petersen S. E. van Mier H. Fiez J. A. Raichle M. E. (1998). The effects of practice on the functional anatomy of task performance. Proc. Natl. Acad. Sci. U.S.A. 95, 853–860. 10.1073/pnas.95.3.853 9448251 PMC33808

[B68] Platt M. L. Glimcher P. W. (1999). Neural correlates of decision variables in parietal cortex. Nature 400, 233–238. 10.1038/22268 10421364

[B69] Pochon J. B. Levy R. Poline J. B. Crozier S. Lehericy S. Pillon B.. (2001). The role of dorsolateral prefrontal cortex in the preparation of forthcoming actions: an fMRI study. Cereb. Cortex 11, 260–266. 10.1093/cercor/11.3.260 11230097

[B70] Poldrack R. A. Prabhakaran V. Seger C. A. Gabrieli J. D. (1999). Striatal activation during acquisition of a cognitive skill. Neuropsychology 13, 564–574. 10.1037/0894-4105.13.4.564 10527065

[B71] Preuschoff K. Bossaerts P. Quartz S. R. (2006). Neural differentiation of expected reward and risk in human subcortical structures. Neuron 51, 381–390. 10.1016/j.neuron.2006.06.024 16880132

[B72] Rao L. L. Li S. Jiang T. Zhou Y. (2012). Is payoff necessarily weighted by probability when making a risky choice? Evidence from functional connectivity analysis. PLoS One 7:e41048. 10.1371/journal.pone.0041048 22815908 PMC3398869

[B73] Sakai K. (2008). Task set and prefrontal cortex. Annu. Rev. Neurosci. 31, 219–245. 10.1146/annurev.neuro.31.060407.125642 18558854

[B74] Salzman C. D. Belova M. A. Paton J. J. (2005). Beetles, boxes and brain cells: neural mechanisms underlying valuation and learning. Curr. Opin. Neurobiol. 15, 721–729. 10.1016/j.conb.2005.10.016 16271457 PMC2398703

[B75] Saunders E. M. (1993). Stock prices and wall street weather. Am. Econ. Rev. 83, 1337–1345.

[B76] Schiebener J. Brand M. (2015). Decision making under objective risk conditions-a review of cognitive and emotional correlates, strategies, feedback processing, and external influences. Neuropsychol. Rev. 25, 171–198. 10.1007/s11065-015-9285-x 25894847

[B77] Schultz W. (2008). Introduction. Neuroeconomics: the promise and the profit. Philos. Trans. R. Soc. B Biol. Sci. 363, 3767–3769. 10.1098/rstb.2008.0153 18829431 PMC2581782

[B78] Sescousse G. Caldú X. Segura B. Dreher J. C. (2013). Processing of primary and secondary rewards: a quantitative meta-analysis and review of human functional neuroimaging studies. Neurosci. Biobehav. Rev. 37, 681–696. 10.1016/j.neubiorev.2013.02.002 23415703

[B79] Shallice T. (2004). The fractionation of supervisory control, in The Cognitive Neuroscience, ed Gazzaniga M. S. (Cambridge, MA: MIT Press), 943–956.

[B80] Smith V. L. Walker J. M. (1993). Monetary rewards and decision cost in experimental economics. Econ. Inq. 31, 245–261. 10.1111/j.1465-7295.1993.tb00881.x

[B81] Sugam J. A. Saddoris M. P. Carelli R. M. (2014). Nucleus accumbens neurons track behavioral preferences and reward outcomes during risky decision making. Biol. Psychiatry 75, 807–816. 10.1016/j.biopsych.2013.09.010 24143880 PMC3992205

[B82] Vlaev I. (2012). How different are real and hypothetical decisions? Overestimation, contrast and assimilation in social interaction. J. Econ. Psychol. 33, 963–972. 10.1016/j.joep.2012.05.005

[B83] Vogt S. Buccino G. Wohlschlager A. M. Canessa N. Shah N. J. Zilles K.. (2007). Prefrontal involvement in imitation learning of hand actions: effects of practice and expertise. Neuroimage 37, 1371–1383. 10.1016/j.neuroimage.2007.07.005 17698372

[B84] Weickert T. Goldberg T. Callicott Q. C. Apud J. Das S. Zoltick B.. (2009). Neural correlates of probabilistic category learning in patients with schizophrenia. J. Neurosci. 29, 1244–1254. 10.1523/JNEUROSCI.4341-08.2009 19176832 PMC2775494

[B85] Yang T. Shadlen M. N. (2007). Probabilistic reasoning by neurons. Nature 447, 1075–1080. 10.1038/nature05852 17546027

[B86] Zalocusky K. A. Ramakrishnan C. Lerner T. N. Davidson T. J. Knutson B. Deisseroth K. (2016). Nucleus accumbens D2R cells signal prior outcomes and control risky decision-making. Nature 531, 642–646. 10.1038/nature17400 27007845 PMC5717318

